# Evaluating the role of indoor environmental quality in predicting ocular and general sick building syndrome: insights from the AIRMED project

**DOI:** 10.7717/peerj.21489

**Published:** 2026-07-21

**Authors:** Kampanat Wangsan, Vithawat Surawattanasakul, Wachiranun Sirikul, Ratana Sapbamrer

**Affiliations:** 1 Department of Community Medicine, Faculty of Medicine, Chiang Mai University, Chiang Mai, Thailand; 2 Environmental and Occupational Medicine Excellence Center, Faculty of Medicine, Chiang Mai University, Chiang Mai, Thailand

**Keywords:** Indoor environmental quality, Sick building syndrome, Screen time, Formaldehyde, Ergonomics

## Abstract

**Background:**

Modern buildings, designed for energy efficiency often lead to poor indoor air quality (IAQ), combined with prolonged indoor working activity resulting in Sick Building Syndrome (SBS) symptoms among occupants. This study aimed to assess the prevalence of SBS symptoms especially ocular and general symptoms and their association with IAQ and ergonomic factors among office workers in a healthcare facility.

**Method:**

A cross-sectional study involving 261 office workers was conducted, assessing demographics, working conditions, and SBS symptoms using questionnaires. Indoor Environmental Quality (IEQ) parameters were measured, including thermal comfort, chemical, and biological parameters, light, and building inspection.

**Result:**

Results revealed a substantial prevalence of ocular (27.2%) and general (22.2%) SBS symptoms, associated with prolonged computer usage and elevated formaldehyde levels. Multivariable analysis revealed that every hour of increase in computer screen time beyond seven hours increased the risk of ocular and general SBS by 21% and 23%, respectively. Formaldehyde concentrations ≥ ocular 0.74 ppm increased the probability of ocular SBS by 5.9 times. Furthermore, repetitive wrist and shoulder movements increased the risk of general SBS by 2.48 times.

**Conclusion:**

Recommendations include optimizing IAQ, reducing formaldehyde emissions, and implementing ergonomic workstation strategies to mitigate SBS effects. This study emphasizes the importance of comprehensive workplace interventions for healthier indoor environments and improved occupational health and productivity.

## Introduction

In contemporary society, individuals spend more than 90% of their daily lives within indoor environments, making indoor air quality (IAQ) a critical determinant of public health (Redlich, Sparer & Cullen, 1997; Sahlberg et al., 2013). In the pursuit of energy efficiency and operational cost reductions, modern architectural designs increasingly utilize airtight building envelopes and recirculating heating, ventilation, and air conditioning (HVAC) systems to minimize thermal exchange (Olatunde et al., 2024). While these engineering strategies support environmental sustainability, they often inadvertently result in suboptimal ventilation rates and the sequestration of indoor air pollutants (IAPs) (Norbäck et al., 2017; Ye et al., 2017). Consequently, prolonged occupancy in such environments is frequently associated with “Sick Building Syndrome” (SBS), a complex of nonspecific symptoms including cephalgia, mucosal irritation of the ocular and respiratory tracts, dermal pruritus, and persistent ocular fatigue (Joshi, 2008; Redlich, Sparer & Cullen, 1997; Wolkoff, 2013). These symptoms typically lack a single identifiable clinical etiology and manifest predominantly among specific cohorts of building occupants (Magnavita, 2015; Marmot et al., 2006). SBS happen while staying in the building and usually resolve soon after leaving, and are believed to be associated with IAPs and individual susceptibility to low concentrations of contaminant (Joshi, 2008; Occupational Safety and Health Administration, 1999). The World Health Organization has raised this concern since the 1980s and estimated that 30% of newly renovated buildings may face SBS. Encountering SBS led to substantially disturbing people’s performance, and relationships resulting in a loss of productivity (EPA, 1991). Although the cause of SBS is still unclear, there are several related factors including chemical or biological contaminants, poor ventilation, psycho-social factors, IAQ, inappropriate lighting, and ergonomic design (Joshi, 2008).

The common SBS symptoms included mucous-membrane irritation (eye, throat irritation), neurotoxic effects, (headache, fatigue, poor concentration), respiratory symptoms (shortness of breath, cough, wheezing), skin symptoms (rash, itching, dryness), and chemosensory changes (abnormal odor sensation, visual disturbance) (Redlich, Sparer & Cullen, 1997). Different SBS symptoms may be related to different factors; for example, ocular symptoms are likely related to chemical contaminants (Norbäck et al., 2017; Wolkoff, 2013) Respiratory symptoms may relate to humidity and temperature (Surawattanasakul et al., 2022), or general symptoms could relate to stress at work (Andersson et al., 1999; Lahtinen, Sundman-Digert & Reijula, 2004; Magnavita, 2015; Marmot et al., 2006). Understanding the characteristics of clinical SBS symptoms would be beneficial to optimizing the IAQ to reduce SBS. Office workers spend most of their time inside buildings. Besides, the working environment in the office may be related to SBS, such as chemical contaminants from office equipment, copy machines, and building materials (Occupational Safety and Health Administration, 1999), improper lighting, prolonged computer display usage with poor ergonomics design, inadequate ventilation, or stress at work (Redlich, Sparer & Cullen, 1997). Volatile Organic Compounds (VOCs), especially formaldehyde, which are usually released from building materials, have been reported as being related to SBS in the office (Sahlberg et al., 2013; Takeda et al., 2009).

However, limited research has been undertaken to evaluate indoor environmental quality (IEQ) and SBS among administrative staff in healthcare facilities that operate around the clock, compounded by ongoing facility renovations, making IAQ monitoring and management challenging. Understanding and managing factors contributing to SBS are paramount for enhancing the health and productivity of workers.

As summarized in Table 1, while previous studies have extensively explored the role of psychosocial factors (Lahtinen, Sundman-Digert & Reijula, 2004; Marmot et al., 2006) and chemical contaminants (Norbäck et al., 2017; Takeda et al., 2009), there is an increasing shift towards using smart sensor technologies and mixed-method approaches (Awolesi, 2025; Leccese et al., 2025). Our current study builds upon this by focusing specifically on the correlation between real-time monitored physical parameters and ocular-specific symptoms among office workers, bridging the gap between physical monitoring and subjective stakeholder perceptions. Consequently, this study aims to establish the prevalence of ocular and general symptoms associated with SBS among office personnel and their correlation with IEQ.

**Table 1:** Summary of literature review on indoor environment and Sick Building Syndrome (SBS).

**Author (Year)**	**Focus/Scope**	**Methodology**	**Key Findings**
**Redlich, Sparer & Cullen (1997)(1)**	General overview of SBS	Literature Review	Identified multi-factorial causes including chemical, biological, and psychosocial factors.
**Wolkoff (2013) (7)**	Health coutcome & IAQ	Review	Office VOC level rarely reach irritation thresholds; discomfort stems mainly from ventilation, CO2, humidity, and secondary pollutions, with minimal evidence of long-term health effects.
**Norbäck et al. (2017) (5)**	VOCs, NO2, and SBS in schools	Cross-sectional/Air sampling	Significant associations between formaldehyde/VOCs and ocular/nasal symptoms.
**Surawattanasakul et al. (2022) (12)**	Respiratory/Skin SBS (AIRMED Project)	Mixed-method (Sensors + Survey)	PM2.5 and CO2 levels in hospitals were linked to respiratory and skin issues.
**Magnavita (2015) (8)**	Occupational health & SBS	Long-term observational	SBS is a complex puzzle involving both physical environment and personal stress.
**Marmot et al. (2006) (9)**	Psychosocial factors in SBS	Epidemiological (Whitehall II)	Psychosocial work environment (low control) is a stronger predictor than physical environment.
**Lahtinen, Sundman-Digert & Reijula (2004) (13)**	Diagnosis of indoor problems	Questionnaire-based	Psychosocial climate is crucial for diagnosing and resolving indoor air complaints.
**Andersson et al. (1999) (14)**	School personnel & SBS	Survey/Physical monitoring	Combined effect of physical (ventilation) and psychosocial factors on symptoms.
**Takeda et al. (2009) (15)**	New dwellings and SBS	Environmental monitoring	Formaldehyde and VOCs in new buildings significantly increase SBS risk.
**Sahlberg et al. (2013) (2)**	Molds, bacteria, and MVOCs	Indoor air sampling	Airborne molds and plasticizers in homes are related to increased SBS prevalence.
**Awolesi, Ghafari & Reams (2025) (18)**	IAQ & Health in Office Buildings	Comprehensive Review	Highlighted the role of advanced ventilation and sensor-based monitoring.
**Leccese et al. (2025) (17)**	Smart Building Systems	Simulation/Experimental	Integration of IoT sensors can optimize IAQ and reduce occupant health risks.
Awolesi, Ghafari & Reams (2025) (16)	Indoor Environmental Quality (IEQ) and energy performance	A multi-method approach	LEED Silver buildings generally demonstrated better IEQ; however, issues such as high humidity, excessive noise, and elevated formaldehyde concentrations were still observed.
**Current Study**	**Ocular and SBS in Office Settings**	**Mixed-method (Sensors + Survey)**	**Integrates real-time physical data with subjective ocular/SBS perceptions.**

## Materials and Methods

### Study design and participants

**Figure 1:**
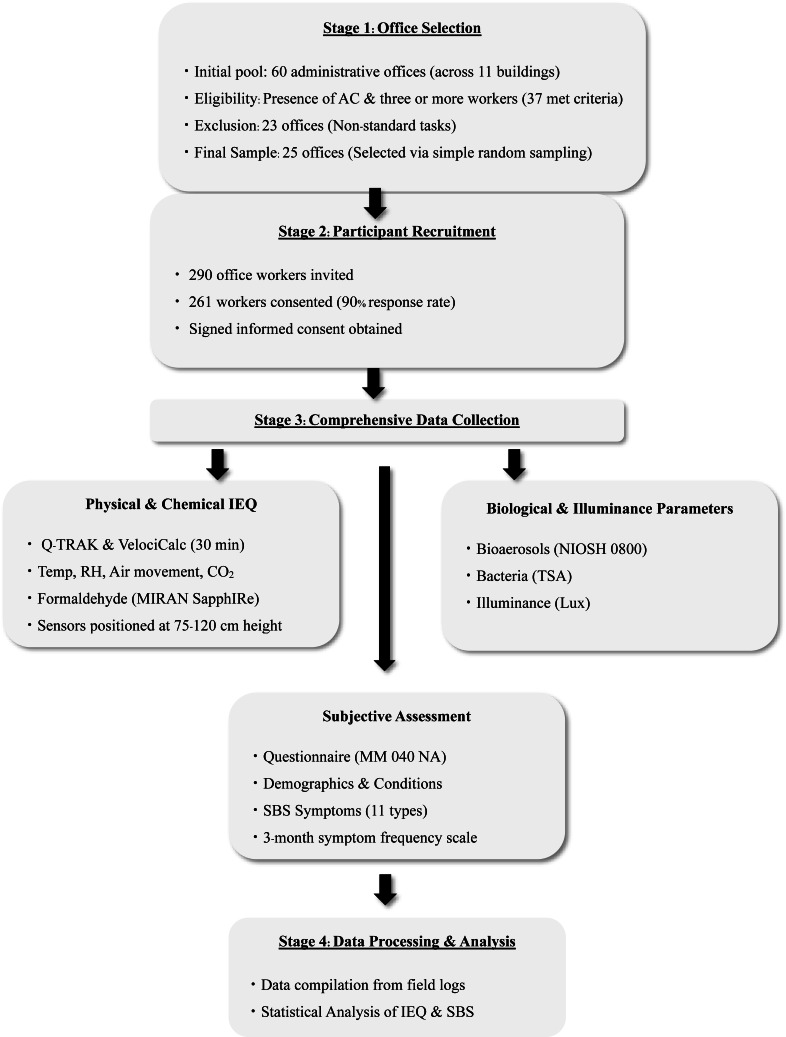
Research workflow diagram of the study. The four primary stages of the investigation: (1) Systematic selection and simple random sampling of office units; (2) Participant recruitment and the informed consent process; (3) Multidimensional data collection, encompassing physical, chemical, and biological IEQ monitoring alongside subjective symptom assessments; and (4) Final data processing and statistical analysis.

A cross-sectional study was carried out as part of the AIRMED project, which aims to explore the health impacts of IEQ on office workers within the university. Initially, we acquired a list of all offices and their respective workforce sizes. Offices equipped with air conditioning and employing at least three workers were considered eligible for inclusion. Out of a total of 60 administrative offices spanning 11 buildings, 37 met the inclusion criteria. Subsequently, 23 offices were excluded because their workers primarily performed non-standard office tasks. Using a one-stage cluster sampling approach, 25 offices were selected from the 37 eligible rooms through simple random sampling (random number generator). All 290 office workers within these 25 selected offices were then invited to participate in the study *via* email, announcements, and face-to-face invitations. Participants were requested to complete a self-administered questionnaire within two weeks following the assessment of their working environment. The questionnaire encompassed three sections, which investigated worker demographics, working conditions, and experiences of SBS. All participants provided written informed consent before participating in the study. Of the 290 workers approached, 261 (90.0%) consented to partake in the survey and duly completed the self-reported questionnaire. Figure 1. Research Workflow Diagram of the Study. The flow illustrates the four primary stages of the investigation: (1) Systematic selection and randomization of office units; (2) Participant recruitment and informed consent process; (3) Multi-dimensional data collection involving physical, chemical, and biological IEQ monitoring alongside subjective symptom assessments; and (4) Final data processing and statistical analysis.

### Questionnaires

Participant demographics included age (years), gender (male/female), smoking status (non-smoker/ex-smoker/current smoker), working experience (years), eye equipment (wearing glasses/contact lens), underlying diseases(yes/no) including pterygium/pinguecula, allergic rhinitis, chronic musculoskeletal pain, migraine, and perceptions of the working environment. Participants were asked about their sensitivity to smoke and chemical agents (yes/no). To assess working conditions. Participants were asked about their tasks, regular work hours per week, overtime hours per week at their offices, computer usage (yes/no), and screen times (hours/day), repetitive movement of wrist and shoulder (yes/no), and awkward postures during work (yes/no).

The SBS symptom assessment utilized questions adapted from the standardized Möljömedicin questionnaire (MM 040 NA) for workplaces (Andersson, 1998). This validated questionnaire, designed for epidemiological assessment of IAQ issues in workplaces, consists of three main categories of symptoms: dermal, mucosal, and general, including perceptions of symptom causation by the working environment. The focus was on ocular and general symptoms, encompassing 11 specific symptoms: ocular symptoms (eye irritation, dry eye, tearing, eye itching, and red eye) and general symptoms (headache, feeling heavy-headedness, dizziness, nausea, fatigue, and difficulties concentrating). Symptom frequency over the previous three months was rated on a five-point Likert scale: 3–5 days per week, 1–2 days per week, 2–3 times per month, once a month, and never. SBS criteria in this study were defined as participants experiencing symptoms for more than one day per week and attributing their symptoms to their working environment. Translation and back-translation of the questionnaire between English and Thai were conducted by bilingual occupational health professionals, with a reliability assessment yielding a Cronbach’s Alpha of 0.938 (Surawattanasakul et al., 2022).

### Building inspection and IEQ evaluation

To evaluate the condition of the buildings, the research team conducted extensive data collection through detailed walk-through inspections and investigations, ensuring that every room was covered. The office spaces varied in size, ranging from approximately 20 m^2^ to 120 m^2^ for individual administrative units, while larger shared spaces reached up to 500 m^2^. All offices were characterized by central or split-type air conditioning systems with standard office furniture and electronic equipment. IAQ measurements were conducted in all 25 selected offices. At least one measurement point was established per room to ensure comprehensive coverage. For larger office spaces, the number of measurement points was determined by floor area, with one additional point allocated for every 500 m^2^ and an outdoor air quality measurement, following the guidelines set by the Singapore Standard Council for IAQ 2021 (Singapore Standard Council, 2021).

The data collection was conducted in October 2021, during the transition from the rainy to the cool-dry season. This specific period was selected as seasonal variations in tropical regions significantly influence indoor air dynamics and occupant health reporting (Awolesi, 2025; Awolesi, Ghafari & Reams, 2025). The study site in Chiang Mai is classified as a Tropical Savanna Climate (Aw), where building energy strategies must balance ventilation with thermal comfort (Khovalyg et al., 2023).

The Q-TRAK™ Indoor Air Quality Monitor (Model 7575, TSI Incorporated, Shoreview, MN, USA) and the VelociCalc^®^ Plus Multi-Function Ventilation Meter (Model 9565, TSI Incorporated, MN, USA) were employed to measure air temperature (AT), relative humidity (RH), air movement (AM), and various chemical parameters, including carbon dioxide (CO2). Formaldehyde levels indoors were assessed using the MIRAN SapphIRe portable interface ambient air analyzer (Model MIRAN SapphIRe XL, Franklin, VA, USA). Measurement results at each sampling point were carefully documented on field data log sheets, and all instruments were calibrated according to the manufacturer’s specifications.

Biological parameters were assessed following the method 0800 of NIOSH for bioaerosol sampling in indoor air (Lonon, 1998). This process involved using a single-stage airborne microbial-variable impactor method with a flow rate of 28.3 L/min for four minutes. Bacteria were then cultured on tryptone soya agar (TSA) media and incubated for 48 h at 35 °C. Airborne fungi and bacteria concentrations were quantified as the number of colony-forming units per cubic meter (CFU/m^3^).

The calibrated light meter “Extech Model 407026” was used to measure the lighting. The measurement protocol was conducted following the ministerial regulation of the Thai Department of Labor Protection and Welfare (Department of Labour Protection and Welfare, 2022). The average light intensity in every 2 × 2 m^2^ area was reported in Lux units. Representative photographs of the office environment and the data collection process are provided in Fig. S1.

**Figure 2:**
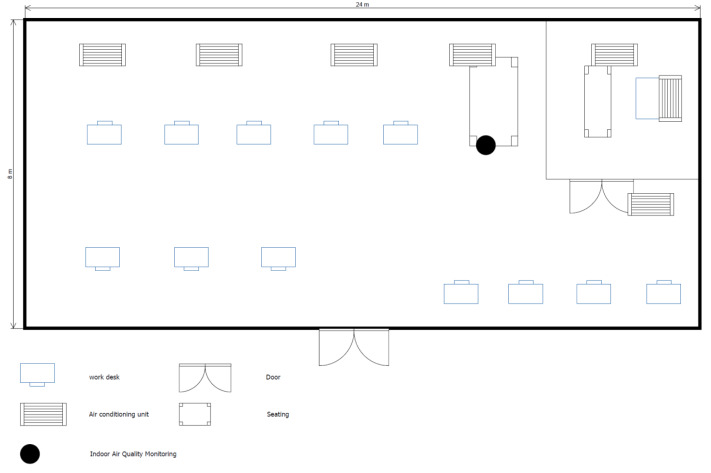
Schematic layout of IEQ monitoring.

The spatial distribution of the monitoring equipment was carefully planned to reflect representative occupant exposure. A schematic of the equipment placement and the temporal monitoring schedule is presented in Fig. 2. all sensors were positioned at least one meter away from walls and windows, and 1.5 m from active HVAC diffusers, at a height corresponding to the occupants’ breathing zone (75–120 cm).

This study followed the Declaration of Helsinki, and the protocol received approval from the Research Ethics Committee of the Faculty of Medicine at Chiang Mai University, Thailand (Study code: COM-2564-08477) All participants provided written informed consent before participating in the study.

### Data analysis

Descriptive analysis was employed to demographically analyze the workers, their working conditions, and IEQ measurements across the 25 offices. Categorical variables were presented as frequencies with percentages, while continuous variables were summarized using means with standard deviations (SD) for parametric data and medians with interquartile ranges (IQR) for non-parametric data. Factors associated with each SBS were identified through exploratory analysis using multivariable logistic regression. Independent factors, including potential associated factors and confounders, were predefined and selected based on established associations with SBS from previous literature and biological plausibility. IEQ parameters were included as continuous data with rescaling to detect clinically significant levels, except for formaldehyde, which was treated as non-parametric data. Non-parametric data were transformed into categorical levels using quartiles to ensure the interpretability of the multivariable analysis results. These variables were incorporated into the final model to derive independent and unbiased magnitudes of association, reported as adjusted odds ratios (aOR) with 95% confidence intervals (95% CI). As our study setting consists entirely of an air-conditioned room some factors will be considered as controlled if their variations were settled in identical conditions, particularly AT, humidity, sensitivity to chemical and smoke, eye equipment, and underlying conditions associated with ocular or general SBS. The confounding factors Statistical analyses were conducted using STATA16 software, and all tests were two-tailed with a significance level set at *p* < 0.05. The findings of the study were reported according to the Strengthening the Reporting of Observational Studies in Epidemiology (STROBE) guidelines (Von Elm et al., 2008).

## Result

A total of 261 office workers participated in the study. Among these, 71 participants (27.2%) reported Ocular SBS symptoms, while 58 participants (22.2%) reported General SBS symptoms. The mean age of the total study population was 40.0 ± 11.2 years. When categorized by symptom type, the mean age for the Ocular SBS group was 37.9 ± 10.9 years, and 35.3 ± 9.4 years for the General SBS group. Female workers constituted the majority of both the Ocular SBS group (*n* = 52; 73.2%) and the General SBS group (*n* = 38; 65.5%). The number of current smokers among and SBS were 1.4% and 5.2%, respectively. The average working experience was seven years in ocular and five years in general SBS. The proportion of participants who use eye equipment was 43.7% in oculars and 29.0% in general SBS. The participants who have health conditions were 45.1% in ocular SBS and 41.4% in general SBS and the most common underlying disease in both groups is allergic rhinitis. At least 70% of participants from both groups reported feeling sensitive to smoke and chemicals in the workplace. Both groups reported around 39 working hours per week. Almost all workers use a computer for their tasks. Computer screen usage times in both groups were seven hours per day, which is around 90% of total working hours. There was a high prevalence of wrist and shoulder repetitive movement in both groups (81.7% and 91.4%. The awkward posture was also a high occurrence, prominently in general SBS (75.9%). The demographic characteristics and occupational profiles of the participants, including their specific working conditions, are summarized in Table 2.

**Table 2:** Participants demographics and working conditions.

Participants demographic	Ocular SBS (*n* = 71)		General SBS (*n* = 58)	
	***n***	**(%)**	***n***	**(%)**
Age (year), mean (±SD)	37.9	(±10.9)	35.3	(±9.4)	
Gender				
Male	19	(26.7)	20	(34.5)	
Female	52	(73.2)	38	(65.5)	
Working year, median (IQR)^a^	7	(2–15)	5	(2–14)	
Smoking				
Non-smoker	68	(95.7)	53	(91.4)	
Ex-smoker	2	(2.8)	2	(3.4)	
Current-smoker	1	(1.4)	3	(5.2)	
Eye equipment	31	(43.7)	29	(50)	
Underlying disease	32	(45.1)	24	(41.4)	
Pterygium/Pinguecula	2	(2.9)	1	(1.7)	
Allergic rhinitis	12	(16.9)	7	(12.1)	
Migraine	4	(5.7)	5	(8.6)	
Chronic Musculoskeletal pain	7	(9.9)	5	(8.6)	
Feeling sensitive to smoke	64	(90.1)	55	(94.8)	
Feeling sensitive to chemical agents	50	(70.4)	46	(79.3)	
Working conditions				
Regular working hour per week, mean (±SD)	39	(±6.0)	39.8	(±6.9)	
Overtime working hour per week, median (IQR)^a^	4	(0–5)	3	(1–5)	
Using computers	71	(100)	57	(98.3)	
Computer screen times (hour per day), mean (±SD)	7	(±2.0)	7.2	(±2.0)	
Repetitive movements of wrist and shoulder	58	(81.7)	53	(91.4)	
Awkward postures	47	(66.2)	44	(75.9)	

**Notes.**

a non-parametric data are presented using median (IQR).

Abbreviations IQR Interquartile range SD Standard deviation

**Figure 3:**
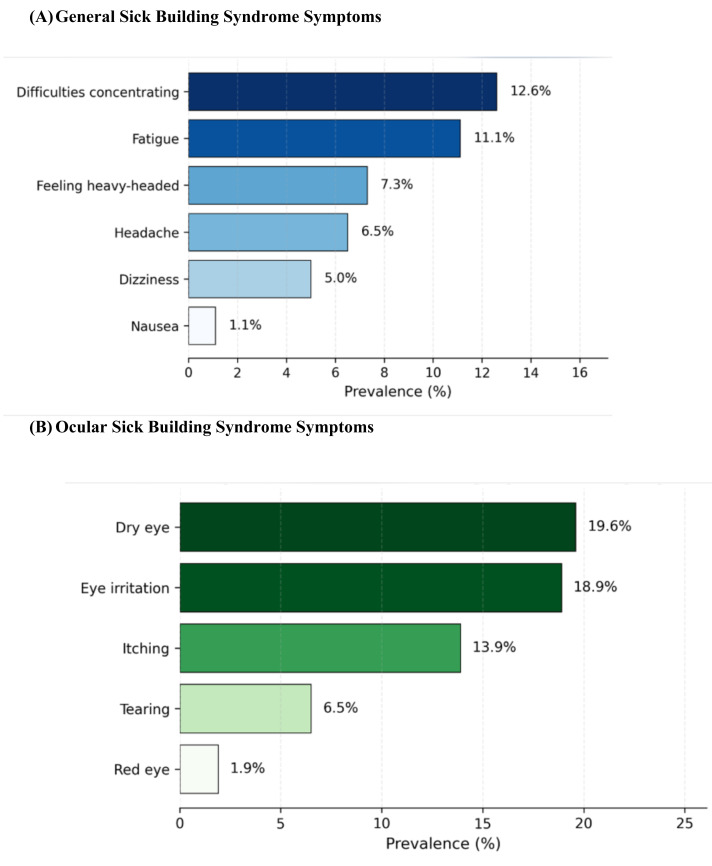
The symptom prevalence of ocular and general sick building syndrome.

The prevalence of symptoms associated with General and Ocular SBS is shown in Fig. 3. For General SBS, the most frequently reported symptom is difficulties concentrating, affecting 12.6% of the 58 individuals surveyed, followed by fatigue (11.1%), feeling heavy-headed (7.3%), headache (6.5%), dizziness (5.0%), and nausea (1.1%). For Ocular-SBS, the prevalence of symptoms among 71 individuals indicates that dry eye is the most common symptom, reported by 19.6% of participants. This is followed by irritation (18.9%), itching (13.9%), tearing (6.5%), and red eye (1.9%).

Assessing air-quality parameters in the working environment across 25 locations provides an overview of thermal comfort and chemical, biological, and lighting conditions. The average room size was 466.96 m^3^ (±402.83). Thermal comfort parameters indicated a mean AT of (23.39 °C ±1.00), with RH averaging 61.83% (±5.66), and AM at 0.11 m/s (±0.08). Chemical analysis showed carbon dioxide levels at 795.75 ppm ±191.36, and formaldehyde concentrations at a median of 0.36 ppm (IQR 0.28−0.74), with a maximum detected value of 2.58 ppm. Biological evaluations revealed a total viable bacterial count of 40 CFU/m^3^ (±20). The lighting conditions were measured, showing an average environmental lighting of 334.99 Lux (±94.87). These environmental parameters, particularly the elevated formaldehyde levels and microbial counts, are further evaluated against established IAQ standards in the Discussion section.

Workers who spend more hours in front of screens daily are notably more susceptible to Ocular SBS symptoms. Specifically, for each additional hour of screen time per day, the odds of experiencing these symptoms increase by 21% (aOR = 1.21, 95% CI [1.00–1.45], *p*-value = 0.049). Another related factor is the presence of formaldehyde in the work environment. Workers exposed to formaldehyde concentrations of 0.74 ppm or higher face a higher risk, with the odds nearly six times those of workers in environments with lower concentrations (aOR = 5.90, 95% CI [1.11–31.65], *p*-value = 0.039). Table 3 illustrates the relationships between worker demographics, working conditions, and IEQ parameters as they relate to ocular SBS symptoms.

**Table 3:** Worker demographic, working conditions, indoor environmental quality related to ocular SBS symptoms.

**Variables**	**Ocular SBS symptoms**	***p*** **-value**
	**aOR (95% CI)**	
**Demographics**		
Age (years)	0.99 (0.94 to 1.03)	0.691
Female	0.65 (0.30 to 1.4)	0.232
Working year	0.98 (0.94 to 1.03)	0.475
Current/Ex-smokers	0.45 (0.16 to 1.26)	0.129
Pterygium/Pinguecula	3.04 (0.33–27.7)	0.324
Allergic rhinitis	1.50 (0.59 to 3.8)	0.396
Feeling sensitive to smoke	0.99 (0.29 to 3.35)	0.986
Feeling sensitive to chemical agents	0.90 (0.42 to 1.92)	0.782
**Working conditions**		
Regular working hour per week	1.06 (0.99 to 1.13)	0.062
Computer Screen Time (Hour/day)	**1.21 (1.00 to 1.45)**	**0.049**
Room sizes (m^3^), increasing: 100 m^3^	1.96 (0.89 to 1.26)	0.481
Environmental lighting (Lux)	1.08 (0.82–1.41)	0.805
**Chemical parameters**		
Carbon dioxide (ppm), increasing 100 ppm	1.04 (0.77 to 1.39)	0.805
Formaldehyde (ppm), (classified by Quartiles) Q1, <0.28	Ref.	
Q2, 0.28 to 0.35	0.37 (0.11 to 1.28)	0.117
Q3, 0.36 to 0.73	1.51 (0.35 to 6.54)	0.581
Q4, ≥ 0.74	**5.90 (1.11 to 31.65)**	**0.039**
**Biological parameters**		
Total viable bacterial count (CFU/m^3^), increasing:10 CFU/m^3^	**1.32 (1.024 to 1.71)**	**0.032**

**Notes.**

aOR was obtained from a multivariable logistic regression analysis with adjustment for air temperature, humidity, sensitive to chemical and smoke, eye equipment, underlying diseases (pterygium/pinguecula, allergic rhinitis).

Abbreviation aOR adjusted odds ratio CFU/m3 colony-forming units per cubic meter ppm parts per million Ref. Reference category for multinomial variables Q Quartile

Bold values indicate statistically significant associations (*p* < 0.05).

Extended hours spent in front of a computer screen are significantly associated with an increased risk of General SBS symptoms. Specifically, each additional hour of computer screen time per day raises the odds of experiencing these symptoms by 23% (aOR = 1.23, 95% CI [1.00–1.50], *p*-value = 0.043). Repetitive movements involving the wrist and shoulders are also significantly associated with an increased risk of General SBS symptoms. Workers engaged in these repetitive tasks have more than double the odds of developing General SBS symptoms (aOR = 2.48, 95% CI [1.14–5.36], *p*-value = 0.021). The associations between individual characteristics, environmental factors, and the prevalence of general SBS symptoms are presented in Table 4.

**Table 4:** Worker demographic, working conditions, indoor environmental quality related to general SBS symptoms.

**Variables**	**General SBS symptoms**	***p*** **-value**
	**aOR (95% CI)**	
**Demographics**		
Age (years)	0.94 (0.90 to 0.99)	0.691
Female	1.06 (0.48 to 2.38)	0.879
Working year	0.99 (0.94 to 1.05)	0.475
Eye Equipment	0.97 (0.56 to 1.66)	0.906
Current/Ex-smokers	1.02 (0.45 to 2.32)	0.968
Chronic Musculoskeletal Pain	2.51 (0.61 to 10.24)	0.198
Migraine	3.83 (0.63 to 23.30)	0.145
Feeling sensitive to smoke	2.04 (0.46 to 8.96)	0.345
Feeling sensitive to chemical agents	1.57 (0.68 to 3.63)	0.388
**Working conditions**		
Regular working hour per week	1.07 (0.99 to 1.15)	0.062
Computer Screen Time (hour/day)	**1.23 (1.00 to 1.50)**	**0.043**
Repetitive Wrist and Shoulder Movements	**2.48 (1.14 to 5.36)**	**0.021**
Awkward Working Position	2.78 (0.91 to 8.34)	0.071
Room sizes (m^3^), increasing 100 m^3^	1.02 (0.85 to 1.21)	0.481
**Chemical parameters**		
Carbon dioxide (ppm), increasing 100 ppm	0.96 (0.69 to 1.37)	0.816
Formaldehyde (ppm), (classified by Quartiles) Q1, <0.28	Ref.	
Q2, 0.28 to 0.35	0.73 (0.22 to 2.47)	0.617
Q3, 0.36 to 0.73	0.44 (0.08 to 2.32)	0.332
Q4, ≥ 0.74	0.76 (0.12 to 4.83)	0.769
**Biological parameters**		
Total viable bacterial count (CFU/m^3^), increasing 10 CFU/m^3^	1.05 (0.79 to 1.37)	0.748

**Notes.**

aOR was obtained from a multivariable logistic regression analysis with adjustment for air temperature, humidity sensitive to chemical and smoke, eye equipment, underlying diseases (chronic musculoskeletal pain, migraine).

Abbreviation aOR adjusted odds ratio CFU/m3 colony-forming units per cubic meter ppm parts per million Ref. Reference category for multinomial variables Q Quartile

## Discussion

The current study aimed to find the relationship between IEQ which may be related to SBS, focusing on ocular and general symptoms. Due to most of the building working conditions being office and computer work, we found that ocular and general SBS may be commonly found among those workers and share related factors that could be modified to reduce both symptoms at the same time. In this article, we will discuss each symptom and then combine our ideas later.

### The ocular SBS

The number of ocular SBS in the current study showed 27.2%, which is close to the studies in Somalia, Taiwan, and Sweden (23–28.7%%) (Lu et al., 2017; Nordström, Norbäck & Akselsson, 1995; Yussuf et al., 2023) but higher than the study in Nepal, Slovenia, Greece, Singapore, and Malaysia (7.6–11.9%) (Dhungana & Chalise, 2020; Ooi et al., 1998; Tsantaki et al., 2022; Zainal et al., 2019) and lower than the study in China (53%) (Chang et al., 2015). These different results from different studies could be explained by some factors such as health conditions, working conditions, or IAPs. For health condition factors, some clinical studies on SBS may not notice preexisting conditions where the triggering agent may not directly induce symptoms but rather aggravate preexisting conditions (Chang et al., 1993). Some health conditions may have the same presentation symptoms as ocular SBS such as allergic rhinoconjunctivitis, pterygium, or pingueculas which may have dry eyes or eye irritation. The current study found that 16.9% and 2.9% of participants who had ocular SBS had allergic rhinitis and pterygium/pingueculas respectively, but after adjusted analysis, we could not find statistically significant relations between allergic rhinitis and ocular SBS. Our findings were supported by the previous study that reported the disconnect between the ocular clinical manifestations and the local immunological characteristics observed in tear cytokines, which hints at the possibility that SBS could be distinct from allergic conjunctivitis (Saeki, Kadonosono & Uchio, 2017).

Our study suggests that computer working tasks may increase the risk of ocular SBS. The reason may be that computer workers tend to have a lower blinking rate than in normal activities for 69% (Skotte et al., 2007). The lower blinking rate could lose the moisture needed to maintain the tear film that protects the ocular surface from environmental pollutant exposure (Dilly, 1994). Computer vision syndrome (CVS), which is a common problem among computer workers, could have the same presentation as ocular SBS. The symptoms of computer vision symptom include redness, irritation, or eye pain, which could happen after prolonged computer work without breaks, improper light adjustment, and refractive error (Blehm et al., 2005). Even CVS and SBS could have overlapping symptoms, but the prevention methods are similar by adjusting the proper working environment and reducing the computer working time.

The chemical agents were quite a frequent problem for IAP issues, especially formaldehyde. We found the relation between ocular SBS and formaldehyde concordant with the study in Malaysia, which was conducted in schools (Norbäck et al., 2017). Our findings found the average formaldehyde concentration was 0.36 ppm, which is significantly higher than the Singapore Indoor Air Quality standard, which suggested not over 0.08 ppm (Singapore Standard Council, 2021). Even if the office buildings may not use formaldehyde in working activities, there could be another source of formaldehyde. The high number for formaldehyde must be from other sources including building materials and insulation, household products such as glues, permanent press fabrics, paints and coatings, lacquers and finishes, and paper products (U.S. Environmental Protection Agency, 2023). The significant association found between formaldehyde concentrations (0.74 ppm) and ocular SBS aligns with international benchmarks, as this threshold approaches the OSHA Permissible Exposure Limit (PEL) of 0.75 ppm (Occupational Safety and Health Administration, 2024).

We found a significant correlation between bacterial concentration and ocular symptoms. Specifically, for every 10 CFU/m^3^ increase in total viable bacterial count above the baseline, the odds of having ocular SBS increased by 32% (Adjusted OR 1.32; 95% CI [1.024–1.71]). Interestingly, the Singapore IAQ 2021 recommended that the bacterial concentration in air should be less than 1,000 CFU/m^3^ (Singapore Standard Council, 2021). Thus, our study might be beneficial to developing new IAQ recommendations in the future to prevent ocular SBS from bacteria in the air. It is possible that not only the concentration, but also different microorganism species could cause different ocular symptoms. The study from Malaysia reported that the bacterial genera Izhakiella and an unclassified genus from Euzebyaceae were associated with the ocular symptoms of SBS (Fu et al., 2021). The narrative review from the ophthalmologist predicted that the cause of dry eye, which is the most common ocular SBS symptom, is from an imbalance of homeostasis with microbiome on the ocular surface (Wang et al., 2022). The next study should evaluate the relationship between the ocular flora and the airborne organism, which may help us determine which pathogen is more important to prevent. However, increasing ventilation and decreasing humidity were the keys to reducing the bacterial concentration (Qiu et al., 2022). But the air humidity should not be controlled too low because it could cause more ocular symptoms (Patel et al., 2023).

### The general SBS

In this study, the prevalence of general SBS, defined as the presence of at least one general symptom (such as headache, fatigue, or dizziness), was 22.2%, which is intermediate between the previous studies, ranging from 16.7%–69.3% (Akova et al., 2022; Apte, Fisk & Daisey, 2000; Arikan, Tekin & Erbas, 2018; Dhungana & Chalise, 2020; Kalender Smajlović, Kukec & Dovjak, 2019; Lu et al., 2015; Ooi et al., 1998; Sakellaris et al., 2021; Tsai, Lin & Chan, 2012; Tsantaki et al., 2022; Zainal et al., 2019). We assumed that the wide range of prevalence from previous studies may be due to the nonspecific symptoms of general SBS. The reported symptoms of the SBS are subjective, especially the general SBS, which includes broad-spectrum symptoms (tiredness, feeling heavy-headed, headache, nausea, dizziness, and difficulty concentrating). Additionally, many factors were reported related to general SBS, including health conditions, age, and allergies (Mentese & Tasdibi, 2016; Norbäck, 2009), work conditions; computer display (Skov, Valbjørn & Pedersen, 1990), job stress and social support (Andersson et al., 1999; Lahtinen, Sundman-Digert & Reijula, 2004; Lahtinen, Sundman-Digert & Reijula, 2004), and IAQ; indoor temperature (Norbäck, 2009).

Remarkably, most of the studies about general SBS were conducted in office buildings, and the most common work was computer-based tasks. Our results supported that the computer working increased the odds of having general and ocular SBS. Several studies described that musculoskeletal and ocular problems are very common among computer workers and have been discussed in the term CVS (Blehm et al., 2005). The CVS symptoms are not only ocular or visual, but also related symptoms such as headache and muscle pain (Seguí Mdel et al., 2015). The CVS and SBS may share characteristics or overlapping clinical symptoms, and the affected workers may have the same risk factors. The current study found that longer computer working hours were associated with a higher risk of both ocular and general SBS symptoms. Specifically, each additional hour of screen time exceeding seven hours per day increased the odds of general symptoms by 23% (aOR 1.23; 95% CI [1.00–1.50]). This is consistent with evidence from CVS studies where risk increases by 12% for each hour after an 8.6-hour exposure threshold (Wangsan et al., 2022). Both findings suggest that the risk is cumulative and becomes particularly significant after a prolonged duration of continuous computer usage.

Repetitive wrist and shoulder movements are common physical activities of computer users, and our study revealed that this was related to increasing the risk of general SBS. Several studies reported that prolonged computer usage was associated with muscle pain, especially in the neck and shoulder region (Baba & Daruis, 2016; Smith et al., 2009). Considering the symptoms of general SBS could somewhat result from chronic muscle pain and physiological response. The musculoskeletal pain from computer usage has multifactorial factors and could be triggered by work demands and psychosocial stressors (Griffiths, Mackey & Adamson, 2007). This is concordant with many studies about general symptoms often reported as related to psychosocial factors, and most of the participants were office workers who often use computers (Andersson et al., 1999; Lahtinen, Sundman-Digert & Reijula, 2004; Magnavita, 2015; Marmot et al., 2006). The next study should be conducted to evaluate the pathophysiological response to computer work and SBS.

### Recommendations

We suggest that computer working tasks and high concentrations of formaldehyde have an important relationship to ocular and/or general symptoms. Office work hygiene including both IAP management and computer workstations approaches must be applied in the workplace to reduce the SBS symptoms and consequences.

The IEQ should be evaluated, especially in the room where workers perceived SBS. The optimization of the workplace environment should consider both occupational exposure limits (OEL) and IAQ standards, especially in office rooms without certain chemical sources in the workplace. All IEQ parameters should be optimized following the IAQ standard to lower the SBS. Formaldehyde may be the most common airborne pollutant in offices because the sources of it are building materials, furniture, paint, coating, or some household product (U.S. Environmental Protection Agency, 2023).

Beyond conventional sources such as particleboard furniture, lacquers, and permanent-press fabrics, the elevated formaldehyde levels in this healthcare facility may be exacerbated by rigorous disinfection protocols. As observed in similar institutional settings, medical-grade cleaning products used near workstations can lead to transient but significant spikes in formaldehyde and other VOCs. While these odors may appear transient, persistent exposure, especially in poorly ventilated areas, can lead to chronic respiratory irritation and ocular discomfort. Therefore, we recommend that building managers evaluate the chemical composition of cleaning agents and consider “off-gassing” periods or enhanced ventilation following disinfection cycles to mitigate occupant exposure.

The US EPA introduced the reduction of formaldehyde concentration by three methods (U.S. Environmental Protection Agency, 2023). The first way is reducing formaldehyde emission by using a dehumidifier and air conditioning to control or reduce humidity and maintain a moderate temperature. The second technique is increasing indoor ventilation, especially after bringing in the potential formaldehyde source. The last way is using low-releasing formaldehyde materials, for example, certified composite wood products. The application of IAQ standards should be local or national, which is feasible in different settings. The Green Building Material Project (GBM) in Taiwan was a good example of reducing SBS by using a national policy. GBM is the system that aims to have a sustainable built environment and healthy living quality by giving certification to non-toxic building and decoration materials. Alongside the Indoor Air Quality Management Act (IAQMA), which provides compulsory guidelines to control IAQ, leading to 75% of healthy GBM products dominating the market (Tsai, 2018). The Department of Health under the Ministry of Public Health, Thailand just released the announcement of IAQ standards for public buildings in 2022 (Department of Health MoPH, Thailand, 2022). However, there is still no national policy or guideline for IAQ management. The national guidelines to reduce IAP should be promulgated to help reduce building-related health problems, especially in public buildings.

In addition to traditional workstation adjustments, the implementation of sit-stand workstations (standing desks) has emerged as a promising strategy to mitigate the adverse effects of prolonged sedentary work. Recent evidence suggests that standing desks can encourage more frequent movement and micro-breaks away from the workstation, which may help reduce musculoskeletal discomfort and limit the duration of continuous exposure to indoor air pollutants. By facilitating a more active working posture, these interventions could play a vital role in reducing the overall burden of general SBS symptoms among office workers (Chambers, Robertson & Baker, 2019; Karakolis & Callaghan, 2014).

A healthy computer work strategy should also get attention, together with proper IAQ. A good work policy should focus on both a good workstation and a work schedule. For computer workstation management, there is no single correct computer posture for everyone, but there are various basic settings for a computer workstation depending on body size among the user population. The common factors that most ergonomic standards and guidelines for computer workstations focus on include sitting posture, monitor position, seat, backrest, armrest, work surface, and leg space (Woo, White & Lai, 2016). There are still other environmental factors that correspondingly concern, for example, the proper display and lighting source adjustment to avoid glare or reflection from the screen, and the bothersome noise around the workplace. Work-rest scheduling is also important in reducing SBS. Staying too long in front of the computer display without breaking may cause more stress on the whole body and mind. Providing a relaxation area in the workplace might be a good strategy to reduce work stress and diminish the time exposure to IAP. Finally, implementing a safety office program, including air quality management (Carrer & Wolkoff, 2018; Levasseur et al., 2017), good computer working station design (Rodrigues et al., 2017) and work-rest schedule may be the key to relief of ocular and general sick building syndrome (Balci & Aghazadeh, 2003; Jacques et al., 2018).

## Limitations

The first limitation was that we conducted a cross-sectional study which could not define the causal relation; the next cohort study may evaluate more causal relationships. Secondly, the psychological factors have not been well explored in our study, which should be done in the next study, especially on general SBS. Lastly, the source of formaldehyde was also not identified in the current study. The next study should evaluate the source and reduction of formaldehyde concentration to lower SBS.

## Conclusion

This study highlights the high prevalence of Sick Building Syndrome in office settings, identifying prolonged computer use and elevated formaldehyde levels as primary predictors of ocular and general symptoms. To mitigate these risks, a holistic workplace intervention is essential. Practically, building managers should prioritize IAQ optimization by addressing formaldehyde sources through enhanced ventilation, “off-gassing” periods, and the use of low-emission materials. Furthermore, integrating ergonomic adjustments, such as sit-stand workstations and standardized computer setups, can effectively reduce sedentary strain and pollutant exposure.

At the policy level, our findings emphasize the need for national guidelines in Thailand, similar to the Green Building Material system, to regulate indoor pollutants in public buildings. Although this cross-sectional study cannot establish causality, it provides a framework for future longitudinal research into source-specific reductions and psychological factors. Ultimately, combining IAQ management with ergonomic policies is crucial for improving occupational well-being and organizational efficiency.

##  Supplemental Information

10.7717/peerj.21489/supp-1 Supplemental Information 1 Raw data

10.7717/peerj.21489/supp-2 Supplemental Information 2 Codebook

10.7717/peerj.21489/supp-3 Supplemental Information 3 Questionnaire

10.7717/peerj.21489/supp-4 Supplemental Information 4 Representative photographs illustrating the study settings and the multi-dimensional environmental assessment process (A) Typical Office Environment: An overview of the administrative office layout used in the study, showing the partitioned workstations, ceiling-mounted ventilation systems, and occupant density during standard working hours. (B) Indoor Air Quality (IAQ) Monitoring: Real-time assessment of physical and chemical parameters using the Q-TRAK™ Indoor Air Quality Monitor. The instrument was positioned at the breathing zone level (approximately 75–120 cm above the floor) to ensure representative exposure data. (C) Bioaerosol Sampling: The research team performing airborne microbial sampling using a single-stage impactor. This procedure was used to quantify concentrations of bacteria and fungi (CFU/m^3^) within the office spaces. (D) Lighting Intensity Measurement: Evaluation of the luminous environment at a worker’s station using a calibrated light meter. Measurements were conducted across a 2x2 m 2 grid to determine average light intensity in Lux, following national occupational welfare regulations.

## Additional Information and Declarations

### Data Availability

The following information was supplied regarding data availability: The data and code are available in the Supplemental Files.
