# Supplementary material for: Evaluating the role of indoor environmental quality in predicting ocular and general sick building syndrome: insights from the AIRMED project
Source: PeerJ. 2026 Jul 21;14:e21489. doi: 10.7717/peerj.21489 (PMC13398389; doi:10.7717/peerj.21489)
Supplement: Supplemental Information 3 [file peerj-14-21489-s003.pdf]

แบบสอบถาม

การศึกษาวิจัยเรื่องความชุกและปัจจัยที่สัมพันธ์กับกับกลุ่มอาการป่วยเหตุอาคาร

1.ข้อมูลทั่วไป

1. เพศ ______(0)หญิง ______(1)ชาย

2. อายุ _______ ปี

3. ตำแหน่งงาน ________________________________

4. ฝ่าย/งาน/ภาควิชา_______________หน่วยย่อย_____________________

5. สถานที่ทำงาน อาคาร__________ ชั้น____ เลขที่ห้องทำงาน_____________

6. ระยะเวลาการทำงานในตำแหน่งปัจจุบัน______ ปี

7. .ใช้อุปกรณ์ช่วยในการมองเห็นหรือไม่

______(0)ไม่ใช้ ______(1)แว่นสายตา ______(2)คอนเทคเลนส์

8. สูบบุหรี่

______(0)ไม่เคยสูบ

______(1)สูบแต่เลิกแล้ว

______(2)ปัจจุบันสูบอยู่

9. โรคที่เป็นปัจจุบัน

______(1)ต้อลม/ต้อเนื้อ _____(2)ภูมิแพ้ทางเดินหายใจ

______(3)ไซนัสอักเสบ ______(4)โรคหืด

______(5)ไมเกรน ______(6)ปวดกล้ามเนื้อเรื้อรัง

______(7)อื่นๆ

<u>2.ข้อมูลเกี่ยวกับอาการ</u> ในช่วงระยะเวลา 3 เดือนที่ผ่านมา ท่านมีอาการเหล่านี้เกิดขึ้น

ความถี่: ทุกครั้ง (3–5 วัน/สัปดาห์), บ่อยครั้ง (1–2 วัน/สัปดาห์), บางครั้ง (2–3 ครั้ง/เดือน), นานๆ ครั้ง(1 ครั้ง/เดือน), ไม่มีอาการ

อาการทางตา

10.ระคายเคืองตา

[ ] ทุกครั้ง [ ] บ่อยครั้ง [ ] บางครั้ง [ ] นานๆ ครั้ง [ ] ไม่มีอาการ |

ท่านคิดว่าอาการดังกล่าว เกิดจากสภาพแวดล้อมการทำงาน [ ] ใช่ [ ] ไม่ใช่

11.ตาแห้ง

[ ] ทุกครั้ง [ ] บ่อยครั้ง [ ] บางครั้ง [ ] นานๆ ครั้ง [ ] ไม่มีอาการ |

ท่านคิดว่าอาการดังกล่าว เกิดจากสภาพแวดล้อมการทำงาน [ ] ใช่ [ ] ไม่ใช่

12.น้ำตาไหล

[ ] ทุกครั้ง [ ] บ่อยครั้ง [ ] บางครั้ง [ ] นานๆ ครั้ง [ ] ไม่มีอาการ |

ท่านคิดว่าอาการดังกล่าว เกิดจากสภาพแวดล้อมการทำงาน [ ] ใช่ [ ] ไม่ใช่

13.คันตา

[ ] ทุกครั้ง [ ] บ่อยครั้ง [ ] บางครั้ง [ ] นานๆ ครั้ง [ ] ไม่มีอาการ |

ท่านคิดว่าอาการดังกล่าว เกิดจากสภาพแวดล้อมการทำงาน [ ] ใช่ [ ] ไม่ใช่

14.ตาแดง

[ ] ทุกครั้ง [ ] บ่อยครั้ง [ ] บางครั้ง [ ] นานๆ ครั้ง [ ] ไม่มีอาการ |

ท่านคิดว่าอาการดังกล่าว เกิดจากสภาพแวดล้อมการทำงาน [ ] ใช่ [ ] ไม่ใช่

อาการระบบประสาท

15.ปวดศีรษะ

[ ] ทุกครั้ง [ ] บ่อยครั้ง [ ] บางครั้ง [ ] นานๆ ครั้ง [ ] ไม่มีอาการ |

ท่านคิดว่าอาการดังกล่าว เกิดจากสภาพแวดล้อมการทำงาน [ ] ใช่ [ ] ไม่ใช่

16.มึนศีรษะ

[ ] ทุกครั้ง [ ] บ่อยครั้ง [ ] บางครั้ง [ ] นานๆ ครั้ง [ ] ไม่มีอาการ |

ท่านคิดว่าอาการดังกล่าว เกิดจากสภาพแวดล้อมการทำงาน [ ] ใช่ [ ] ไม่ใช่

17.เวียนศีรษะ

[ ] ทุกครั้ง [ ] บ่อยครั้ง [ ] บางครั้ง [ ] นานๆ ครั้ง [ ] ไม่มีอาการ |

ท่านคิดว่าอาการดังกล่าว เกิดจากสภาพแวดล้อมการทำงาน [ ] ใช่ [ ] ไม่ใช่

18.คลื่นไส้

[ ] ทุกครั้ง [ ] บ่อยครั้ง [ ] บางครั้ง [ ] นานๆ ครั้ง [ ] ไม่มีอาการ |

ท่านคิดว่าอาการดังกล่าว เกิดจากสภาพแวดล้อมการทำงาน [ ] ใช่ [ ] ไม่ใช่

19.อ่อนล้า อ่อนเพลีย

[ ] ทุกครั้ง [ ] บ่อยครั้ง [ ] บางครั้ง [ ] นานๆ ครั้ง [ ] ไม่มีอาการ |

ท่านคิดว่าอาการดังกล่าว เกิดจากสภาพแวดล้อมการทำงาน [ ] ใช่ [ ] ไม่ใช่

20.ขาดสมาธิในการทำงาน

[ ] ทุกครั้ง [ ] บ่อยครั้ง [ ] บางครั้ง [ ] นานๆ ครั้ง [ ] ไม่มีอาการ |

ท่านคิดว่าอาการดังกล่าว เกิดจากสภาพแวดล้อมการทำงาน [ ] ใช่ [ ] ไม่ใช่

3.ข้อมูลด้านบุคคล

21.ท่านคิดเองตนเองมีความไวต่อกลิ่นบุหรี่หรือไม่

(0)ไม่ใช่ (1)ใช่

22.ท่านเคยพบว่าตนเองมีความไวต่อสารเคมีในอากาศที่ทำงานหรือไม่

(0)ไม่ใช่ (1)ใช่

4.ข้อมูลด้านลักษณะงาน

23.ใน<u>เวลาทำงานปกติ</u> ท่านทำงานอยู่ในห้องทำงานนี้ กี่ชั่วโมง/วัน และกี่วันต่อสัปดาห์

______ ชั่วโมง/วัน ______วัน/สัปดาห์

24.โดยเฉลี่ย<u>หลังหมดเวลาทำงาน</u> ท่านทำงานอยู่ในห้องทำงานโดยเฉลี่ยต่ออีก ชั่วโมง/วัน และกี่วันต่อสัปดาห์

______ ชั่วโมง/วัน ______วัน/สัปดาห์

25.ลักษณะการทำงานของท่านต้องใช้คอมพิวเตอร์หรือไม่

______(0)ไม่ใช้ ______(1)ใช้โดยเฉลี่ยวันละ..........ชั่วโมง

26.ในขณะทำงาน ท่านมีการใช้ท่าทางการเคลื่อนไหวข้อมือหรือไหล่ซ้ำๆหรือไม่

______(0)ไม่มี ______(1)มี

27.ในขณะทำงาน ท่านมีการใช้ท่าทางการทำงานที่ไม่เหมาะสมหรือไม่

______(0)ไม่มี ______(1)มี

Questionnaire

Research Study on the Prevalence and Factors Associated with Sick Building Syndrome (SBS)

Section 1: General Information

1. Sex: __(0) Female __(1) Male

2. Age (years): ___________________________

3. Job title/position: ___________________________

4. Division/Department ___________________________ Subunit ___________________________

5. Workplace: Building ___________________ Floor _____ Office room no. ______________

6. Duration in current position (years): ___________________________

7. Do you use any visual aids? __(0) No __(1) Eyeglasses __(2) Contact lenses

8. Smoking status:
__(0) Never smoked

__(1) Former smoker
__(2) Current smoker

9. Current medical conditions (check all that apply):
__(1) Pinguecula/Pterygium __(2) Allergic rhinitis __(3) Sinusitis __(4) Asthma
__(5) Migraine __(6) Chronic myalgia __(7) Other: _____________________

Section 2: Symptoms (in the past 3 months)

Frequency scale: Every time (3–5 days/week) Often (1–2 days/week) Sometimes (2–3 times/month) Rarely (once/month)

Eye symptoms:

10. Eye irritation
[ ] Every time [ ] Often [ ] Sometimes [ ] Rarely [ ] Never | Caused by work? [ ] Yes [ ] No

11. Dry eyes
[ ] Every time [ ] Often [ ] Sometimes [ ] Rarely [ ] Never | Caused by work? [ ] Yes [ ] No

12. Watery eyes/tearing
[ ] Every time [ ] Often [ ] Sometimes [ ] Rarely [ ] Never | Caused by work? [ ] Yes [ ] No

13. Itchy eyes
[ ] Every time [ ] Often [ ] Sometimes [ ] Rarely [ ] Never | Caused by work? [ ] Yes [ ] No

14. Red eyes
[ ] Every time [ ] Often [ ] Sometimes [ ] Rarely [ ] Never | Caused by work? [ ] Yes [ ] No

Nervous system symptoms:

15. Headache
[ ] Every time [ ] Often [ ] Sometimes [ ] Rarely [ ] Never | Caused by work? [ ] Yes [ ] No

16. Light-headedness
[ ] Every time [ ] Often [ ] Sometimes [ ] Rarely [ ] Never | Caused by work? [ ] Yes [ ] No

17. Dizziness/vertigo
[ ] Every time [ ] Often [ ] Sometimes [ ] Rarely [ ] Never | Caused by work? [ ] Yes [ ] No

18. Nausea
[ ] Every time [ ] Often [ ] Sometimes [ ] Rarely [ ] Never | Caused by work? [ ] Yes [ ] No

19. Fatigue/tiredness
[ ] Every time [ ] Often [ ] Sometimes [ ] Rarely [ ] Never | Caused by work? [ ] Yes [ ] No

20. Poor concentration at work
[ ] Every time [ ] Often [ ] Sometimes [ ] Rarely [ ] Never | Caused by work? [ ] Yes [ ] No

Section 3: Personal Factors

21. Do you think you are sensitive to the smell of cigarette smoke? [ ] No [ ] Yes

22. Have you noticed that you are sensitive to airborne chemicals at your workplace? [ ] No [ ] Yes

Section 4: Work Characteristics

23. During normal working hours, how long do you work in this office?
______ hours/day ______ days/week

24. On average after regular hours, how much additional time do you work in this office?
______ hours/day ______ days/week

25. Does your job require a computer? __(0) No __(1) Yes, on average ______ hours/day

26. Do you perform repetitive wrist or shoulder movements while working? __(0) No __(1) Yes

27. Do you work in awkward/poor postures? __(0) No __(1) Yes
