# Supplementary figures and images for: Evaluating the role of indoor environmental quality in predicting ocular and general sick building syndrome: insights from the AIRMED project
Source: PeerJ. 2026 Jul 21;14:e21489. doi: 10.7717/peerj.21489 (PMC13398389; doi:10.7717/peerj.21489)

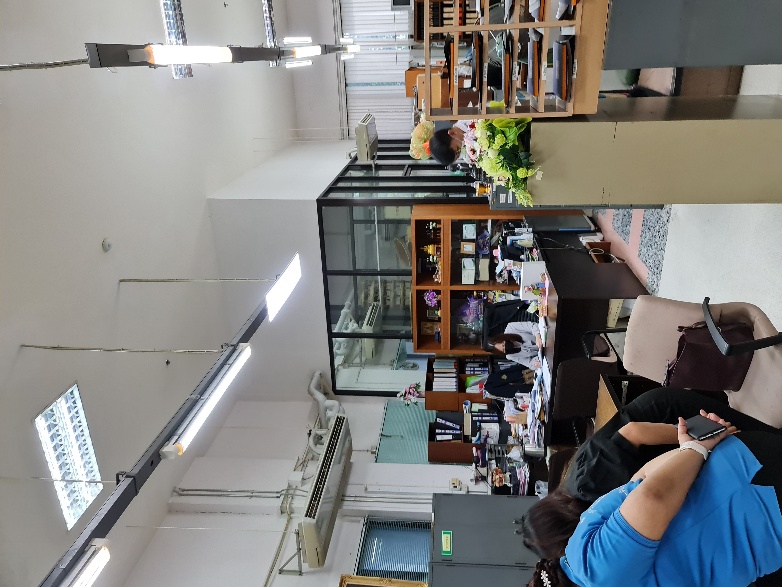


**A**

**C**


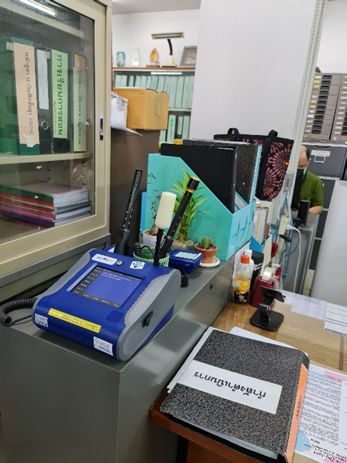


**B**

**D**


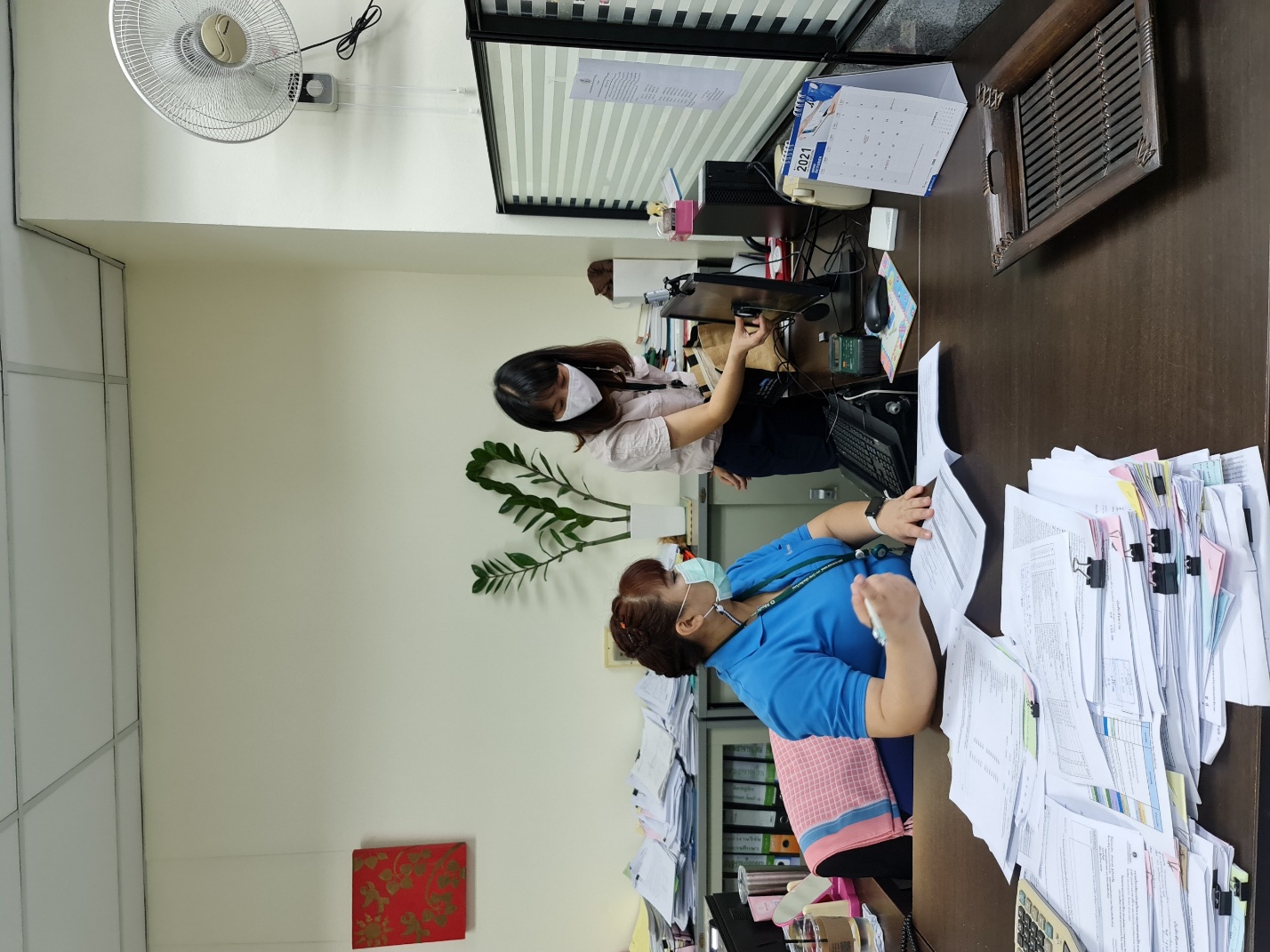


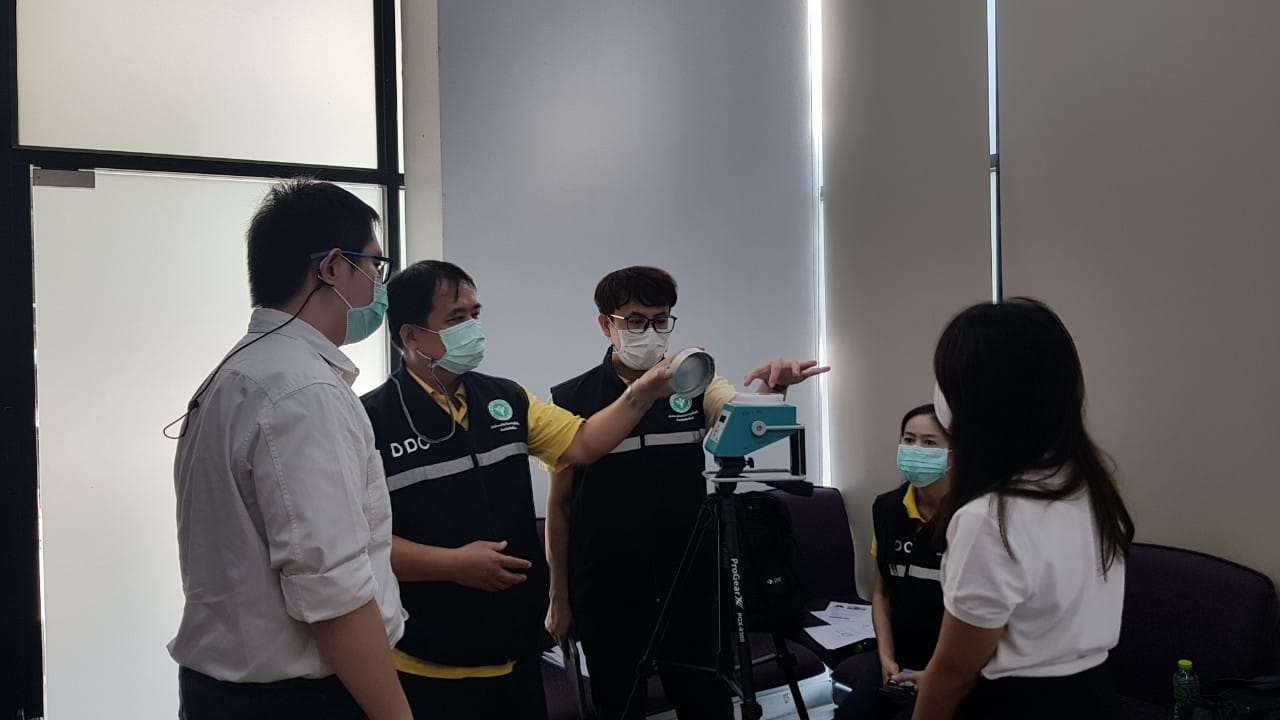

Supplement: Supplemental Information 4 — (A) Typical Office Environment: An overview of the administrative office layout used in the study, showing the partitioned workstations, ceiling-mounted ventilation systems, and occupant density during standard working hours. (B) Indoor Air Quality (IAQ) Monitoring: Real-time assessment of physical and chemical parameters using the Q-TRAK™ Indoor Air Quality Monitor. The instrument was positioned at the breathing zone level (approximately 75–120 cm above the floor) to ensure representative exposure data. (C) Bioaerosol Sampling: The research team performing airborne microbial sampling using a single-stage impactor. This procedure was used to quantify concentrations of bacteria and fungi (CFU/m3) within the office spaces. (D) Lighting Intensity Measurement: Evaluation of the luminous environment at a worker’s station using a calibrated light meter. Measurements were conducted across a 2x2 m 2 grid to determine average light intensity in Lux, following national occupational welfare regulations. [file peerj-14-21489-s004.docx]
